# Risk prediction model for lung cancer incorporating metabolic markers: Development and internal validation in a Chinese population

**DOI:** 10.1002/cam4.3025

**Published:** 2020-04-06

**Authors:** Zhangyan Lyu, Ni Li, Shuohua Chen, Gang Wang, Fengwei Tan, Xiaoshuang Feng, Xin Li, Yan Wen, Zhuoyu Yang, Yalong Wang, Jiang Li, Hongda Chen, Chunqing Lin, Jiansong Ren, Jufang Shi, Shouling Wu, Min Dai, Jie He

**Affiliations:** ^1^ Office of Cancer Screening National Cancer Center/National Clinical Research Center for Cancer/Cancer Hospital Chinese Academy of Medical Sciences and Peking Union Medical College Beijing China; ^2^ Department of Oncology Kailuan General Hospital Tangshan China; ^3^ Health Department of Kailuan (Group) Tangshan China; ^4^ Department of Thoracic Surgery National Cancer Center/National Clinical Research Center for Cancer/Cancer Hospital Chinese Academy of Medical Sciences and Peking Union Medical College Beijing China

**Keywords:** lung cancer, metabolic markers, prospective study, risk prediction model

## Abstract

**Background:**

Low‐dose computed tomography screening has been proved to reduce lung cancer mortality, however, the issues of high false‐positive rate and overdiagnosis remain unsolved. Risk prediction models for lung cancer that could accurately identify high‐risk populations may help to increase efficiency. We thus sought to develop a risk prediction model for lung cancer incorporating epidemiological and metabolic markers in a Chinese population.

**Methods:**

During 2006 and 2015, a total of 122 497 people were observed prospectively for lung cancer incidence with the total person‐years of 976 663. Stepwise multivariable‐adjusted logistic regressions with *P*_entry_ = .15 and *P*_stay_ = .20 were conducted to select the candidate variables including demographics and metabolic markers such as high‐sensitivity C‐reactive protein (hsCRP) and low‐density lipoprotein cholesterol (LDL‐C) into the prediction model. We used the C‐statistic to evaluate discrimination, and Hosmer‐Lemeshow tests for calibration. Tenfold cross‐validation was conducted for internal validation to assess the model's stability.

**Results:**

A total of 984 lung cancer cases were identified during the follow‐up. The epidemiological model including age, gender, smoking status, alcohol intake status, coal dust exposure status, and body mass index generated a C‐statistic of 0.731. The full model additionally included hsCRP and LDL‐C showed significantly better discrimination (C‐statistic = 0.735, *P* = .033). In stratified analysis, the full model showed better predictive power in terms of C‐statistic in younger participants (<50 years, 0.709), females (0.726), and former or current smokers (0.742). The model calibrated well across the deciles of predicted risk in both the overall population (*P*_HL_ = .689) and all subgroups.

**Conclusions:**

We developed and internally validated an easy‐to‐use risk prediction model for lung cancer among the Chinese population that could provide guidance for screening and surveillance.

## Graphical abstract

The epidemiological risk prediction model generated a C‐statistic of 0.731. Significant improvement in C‐statistics was observed when the full model (C‐statistic = 0.735, *P* = .033) was compared to the epidemiological model, suggesting improved calibration when adding metabolic markers to the epidemiological models.

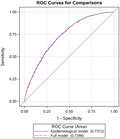

## 1 INTRODUCTION

Lung cancer remains the leading cause of death from cancer worldwide.^1^ In China, lung cancer has been a serious issue in terms of public health. According to the data from the International Agency for Research on Cancer (IARC), in 2018, 37.0% of new cancer cases and 39.2% of cancer‐related deaths occurred in China.^2^ The survival rate of lung cancer was poor (16.1%) in China, however, the prognosis varies greatly at different stages of diagnosis.^3^ The 5‐year survival was <10% for stage IV lung cancer patients, but over 77% for patients with stage I diagnosis.^4^ Taken together, early detection and prevention strategies could have a profound effect on the reduction of the overall disease burden attributable to lung cancer.

It has been shown that lung cancer screening is beneficial. The low‐dose computed tomography (LDCT) screening was shown by the National Lung Screening Trial to reduce lung cancer mortality in asymptomatic high‐risk smokers in 2011.^5^ Then, annual screening for lung cancer with LDCT in adults aged 55‐80 years who were current or former (<15 years since quitting) smokers (≥30 pack‐years) were recommended by the US Preventive Services Task Force (USPSTF).^6^ However, screening using LDCT could lead to a huge number of indeterminate nodules, and a significant proportion of lung cancer cases could not meet the screening entry criteria defined by USPSTF.^7^ Therefore, accurate identification of high‐risk subpopulation to be screened is critical to maximize the efficacy of lung cancer screening.

An accurate lung cancer risk prediction model can contribute effectively to the identification of high‐risk individuals. There have been several lung cancer risk prediction models, primarily on the basis of established risk factors such as smoking, occupational exposures, family history of lung cancer, and respiratory diseases.^8^, ^9^, ^10^, ^11^, ^12^, ^13^, ^14^, ^15^, ^16^, ^17^, ^18^, ^19^, ^20^, ^21^, ^22^, ^23^, ^24^, ^25^, ^26^, ^27^, ^28^, ^29^, ^30^, ^31^, ^32^, ^33^, ^34^, ^35^, ^36^, ^37^, ^38^, ^39^, ^40^ Previous studies have shown that lipids and high‐sensitivity C‐reactive protein (hsCRP) were predictive of lung cancer risk.^41^, ^42^ However, evidence on the predictive performance of these markers in lung cancer beyond smoking‐based epidemiological models is limited. Moreover, there is no risk prediction model for lung cancer among Chinese mainland population based on traditional epidemiological risk factors and biomarkers. Therefore, in the present study, with the focus on established risk factors for lung cancer routinely available in general clinical settings, we aimed to develop and internally validated a risk prediction model for lung cancer.

## 2 METHODS

### 2.1 Study population

The Kailuan cohort is a large prospective dynamic cohort study in Tangshan City, China. The details of the study design and procedure were published previously. In brief, since May 2006, a total of 138 150 thousand employees including retired individuals aged more than 18 years were invited to participate in questionnaire interviews and clinical examinations every 2 years at 11 hospitals that are affiliated with the Kailuan Group.

Participants who provided informed consent and completed the questionnaire interview were enrolled in the present study. Participants with a diagnosis of cancer before the baseline survey (n = 555) or had missing information on covariates included in the models (n = 15 653) were excluded. Ultimately, a total of 122 497 participants were included in the final analysis in this study.

The study was approved by the Medical Ethics Committee of the Kailuan Medical Group. All participants have signed written informed consent forms.

### 2.2 Exposure assessment

Standardized questionnaires and health examination for all individuals were conducted by trained staff at baseline. Information regarding demographics, lifestyle factors, personal medical history, and family history of common noninfectious chronic disease (NCD) as potential indicators were collected. Smoking was defined as smoking ≥1 cigarette per week for at least 12 months. Drinking was defined as drinking ≥1 time per month for at least 6 months. In addition, we derived information on coal dust exposure from each miner's work history.

The weight and height of the individuals were measured on standard stadiometers and scales without wearing shoes. The body mass index (BMI) was calculated by weight (kg)/height (m^2^). The waist circumference (WC) was measured at the midpoint between the supramargin of the iliac crest plane and the lower edge of the rib. The blood pressure (BP) was measured on the left arm using a mercury sphygmomanometer according to the standard recommended procedures.^43^ Systolic blood pressure (SBP) was defined as the point at which the first of two or more Korotkoff sounds are heard, and diastolic blood pressure (DBP) was defined as the disappearance of Korotkoff sound.

We obtained morning fasting venous blood samples of all participants, and then processed and analyzed according to a standard operating procedure. The Hexokinase method was used for the measurement of fasting blood glucose (FBG). The details of the measurement of blood lipids, including total cholesterol (TC), triglycerides (TG), low‐density lipoprotein cholesterol (LDL‐C), and high‐density lipoprotein cholesterol (HDL‐C) have been introduced in previously published studies.^42^

Regarding variables, we assessed potential factors including age (<45, 45‐55, 55‐65, or ≥65 years), gender (male, or female), educational level (illiterate or primary school, junior high school, senior high school, or college and above), status of coal dust exposure (nonexposure or exposure), degree of coal dust exposure (light, moderate, or heavy), status of smoking (never, former, or current), pack‐years of smoking (continuous), duration of smoking (<15, 15‐30, or ≥30 years), age started smoking (<20, or ≥20 years old), smoking cessation time (<15, or ≥15 years), family history of cancer (yes, or no), family history of lung cancer (yes, or no), alcohol intake status (never, former, <1 time per day, or ≥1 time per day), BMI (<18.5, 18.5‐23.9, 24.0‐27.9, or ≥28.0 kg/m^2^), abdominal obesity (men: WC ≥90 cm, women: WC ≥80 cm), FBG (<3.9, 3.9‐5.6, 5.6‐7.0, or ≥7.0 mmol/L), BP (low defined as SBP ≤90 mm Hg or DBP ≤60 mm Hg, normal, or high defined as SBP ≥140 mm Hg or DBP ≥90 mm Hg), TC (quintile), TG (quintile), LDL‐C (quintile), HDL‐C (quintile), and hsCRP.

### 2.3 Ascertainment of lung cancer cases

We followed participants beginning at the baseline examination and ending at the occurrence of cancer, death, or 31 December 2015, whichever event came first. The details of cohort follow‐up and cancer assessment have been published previously.^42^ In brief, people with cancer were identified through biennial health examinations and annual searches of the Tangshan medical insurance system and the Kailuan social security system. Moreover, the outcome information was further confirmed by checking discharge summaries from hospitals where participants were diagnosed or treated. The diagnosis of incident primary lung cancer was confirmed by the reviewed medical records review by clinical experts. Information on pathological diagnosis, imaging diagnosis (including ultrasonography, computerized tomographic scanning, and magnetic resonance imaging), blood biochemical examination, and alpha‐fetoprotein test was collected for the incident lung cancer assessment. Cancers were coded according to the International Classification of Diseases, Tenth Revision (ICD‐10) and lung cancer was coded as C10.

### 2.4 Statistical methods

Categorical variables were described by percentages and the Chi‐squared test was used to compare the difference between different groups. Continuous variables were described by mean (standard deviation) and ANOVA was conducted to compare the difference between different groups. For each risk factor, the association with lung cancer risk was first assessed adjusting for age group by logistic regression. Stepwise multivariable‐adjusted logistic regressions (*P*_entry_ = .15, *P*_stay_ = .20) were conducted to choose the variables included in the prediction model. Odds ratios (ORs) and 95% confidence intervals (CIs) were presented. Predicted risk of lung cancer was calculated by We exp(*β_o_*+∑*β_i_X_i_*)/(1 + exp(β_o_+∑*β_i_X_i_*)), where *β_o_* was the intercept, and *β_i_* was the regression coefficient for risk factor *X_i_*.

Model discrimination was evaluated by receiver‐operating characteristic (ROC) curves and concordance statistics (C‐statistics). In addition, the internal validation of model discrimination was evaluated by 10‐fold cross‐validation. The total cohort was randomly divided into 10 subsets, the prediction model was firstly fitted in 90 percent of the population (training set), and the predictive lung cancer risk was estimated in the remaining 10 percent of the population (validation set). This procedure was repeated for all 10 subpopulations, and the average C‐statistics was calculated. The Hosmer‐Lemeshow goodness‐of‐fit test was used to evaluate the model calibration by comparing the observed and predicted probabilities. A value of *P*_HL_ > .05 indicated satisfactory calibration.

Subgroup analyses were performed by age (<50 years vs ≥50 years), gender (male vs female), and smoking status (never smoking vs former or current smoking).

Furthermore, we calculated the integrated discrimination improvement (IDI) and the net reclassification improvement (NRI) to evaluate the added predictive ability of new factors in risk prediction models.^44^ The NRI focuses on reclassification tables constructed separately for participants with and without events, and quantifies the correct movement in categories—upwards for events and downwards for nonevents.^45^ The IDI focuses on the improvement in the mean discrimination slope and the probability of discrimination between the base model (eg, simple model) and the new models (eg, full model).^45^ Larger NRI and IDI values indicate greater improvements in model discrimination.

In the secondary analysis, we evaluated all the potential predictors among participants aged more than 50 years old, to see the applicability of our model among LDCT screening targeted population. In addition, in sensitivity analyses, continuous variables were also used instead of categorical variables to examine the potential probability of improving discrimination.

All analyses were conducted using the SAS software (Version 9.4; SAS Institute). All statistical tests were two sided, and the significance level was set as *P* < .05.

## 3 RESULTS

### 3.1 Basic characteristics of the study population

A sum of 122 497 participants were enrolled in this study, and the mean age was 50.53 years. The mean levels of BMI, WC, FBG, SBP, DBP, TC, TG, LDL‐C, HDL‐C, HsCRP, and were 24.16 kg/m^2^, 86.78 cm, 5.49 mmol/L, 130.34 mm Hg, 83.48 mm Hg, 190.97 mg/dL, 146.90 mg/dL, 93.58 mg/dL, 58.92 mg/dL, and 2.44 mg/L, respectively. In addition, the rates of tobacco and alcohol intake were 34.70% and 39.09%, respectively (Table 1).

**TABLE 1:** Distribution of baseline characteristics by lung cancer status, Kailuan study, 2006‐2015

Characteristics	Total cohort (n = 122 497)	Lung cancer		*P* value
		Yes (n = 984)	No (n = 121 513)	
Age (years)^a^	50.53 (13.17)	60.00 (10.07)	50.46 (13.16)	<.001
BMI (kg/m^2^)^a^	24.16 (3.27)	23.91 (3.26)	24.16 (3.27)	.019
WC (cm)^a^	86.78 (10.14)	87.76 (9.90)	86.78 (10.14)	.003
FBG (mmol/L)^a^	5.49 (1.69)	5.56 (1.89)	5.49 (1.69)	.251
SBP (mm Hg)^a^	130.34 (21.05)	135.50 (21.52)	130.30 (21.04)	<.001
DBP (mm Hg)^a^	83.48 (11.81)	84.01 (11.89)	83.48 (11.81)	.159
TC (mg/dL)^a^	190.97 (44.53)	193.62 (43.24)	190.95 (44.54)	.062
TG (mg/dL)^a^	146.90 (131.47)	145.06 (116.90)	146.92 (131.58)	.620
LDL‐C (mg/dL)^a^	93.58 (38.58)	91.00 (37.06)	93.60 (38.59)	.036
HDL‐C (mg/dL)^a^	58.92 (18.03)	59.44 (16.03)	58.92 (18.05)	.304
HsCRP (mg/L)^a^	2.44 (6.31)	3.83 (12.27)	2.43 (6.24)	<.001
Gender^b^				
Female	25 695 (20.98)	91 (9.25)	25 604 (21.07)	<.001
Male	96 802 (79.02)	893 (90.75)	95 909 (78.93)	
Education level^b^				
Illiterate or primary school	12 430 (10.15)	192 (19.51)	12 238 (10.07)	<.001
Junior high school	81 169 (66.27)	674 (68.50)	80 495 (66.25)	
Senior high school	18 124 (14.80)	89 (9.04)	18 035 (14.84)	
College and above	10 761 (8.79)	29 (2.95)	10 732 (8.83)	
Smoking status^b^				
Never	79 995 (65.30)	520 (52.85)	79 475 (65.40)	<.001
Former	4044 (3.30)	45 (4.57)	3999 (3.29)	
Current	38 458 (31.40)	419 (42.58)	38 039 (31.30)	
Smoking pack‐years^b^				
<20	18 209 (43.00)	113 (24.35)	18 096 (43.21)	<.001
20‐40	18 088 (42.71)	205 (44.18)	17 883 (42.70)	
≥40	6051 (14.29)	146 (31.47)	5905 (14.10)	
Smoking duration (years)^b^				
<15	6644 (15.69)	30 (6.47)	6614 (15.79)	<.001
15‐30	17 992 (42.49)	132 (28.45)	17 860 (42.64)	
≥30	17 712 (41.48)	302 (65.09)	17 410 (41.57)	
Age start smoking (years old)^b^				
<20	15 866 (37.33)	171 (36.85)	15 695 (37.34)	.831
≥20	26 636 (62.67)	293 (63.15)	26 343 (62.66)	
Smoking cessation duration (years)^b^				
<15	3214 (79.48)	35 (77.78)	3179 (79.49)	.777
≥15	830 (20.52)	10 (22.22)	820 (20.51)	
Alcohol intake status^b^				
Never	74 610 (60.91)	556 (56.50)	74 054 (60.94)	<.001
Former	3587 (2.93)	58 (5.89)	3529 (2.90)	
Current	44 300 (36.16)	370 (37.60)	43 930 (36.15)	
Coal dust exposure status^b^				
Nonexposure	57 784 (47.17)	474 (48.17)	57 310 (47.16)	.529
Exposure	64 713 (52.83)	510 (51.83)	64 203 (52.84)	
Degree of coal dust exposure^b^				
Light	33 680 (52.05)	214 (41.96)	33 466 (52.13)	<.001
Moderate	13 570 (20.97)	120 (23.53)	13 450 (20.95)	
Heavy	17 463 (26.99)	176 (34.51)	17 287 (26.93)	

Abbreviations: BMI, body mass index; DBP, diastolic blood pressure; FBG, fasting blood glucose; HDL‐C, high‐density lipoprotein cholesterol; HsCRP, high‐sensitivity C‐reactive protein; LDL‐C, low‐density lipoprotein cholesterol; SBP, systolic blood pressure; TC, total cholesterol; TG, triglycerides; WC, waist circumference.

^a^ Mean (standard deviation), *P* values from ANOVA.

^b^ N (%), *P* values from the Chi‐squared test.

By December 2015, with a median period of follow‐up of 8.87 (7.09‐9.15) years and a sum of 976 663 person‐years, a total of 984 (0.80%) primary lung cancer cases were identified. Lung cancer cases were typically older, with a lower BMI, lower educational level (junior high school or below), and were more inclined to smoke and drink compared with controls (all *P* < .05). Moreover, the levels of LDL‐C (*P* = .036) and BMI (*P* = .019) were lower in lung cancer cases than controls, while the levels of WC (*P* = .003), SBP (*P* < .001), and HsCRP (*P* < .001) were significantly higher in lung cancer cases than in those without lung cancer (Table 1).

### 3.2 Predictors included in models

Multivariable logistic regression model showed that older age (≥45 years: OR=4.36, 3.25‐5.86; ≥55 years: OR = 7.48, 5.60‐10.01; ≥65 years OR = 13.01, 9.77‐17.57), male (OR = 1.77 1.40‐2.23), smoking status (former smoker: OR = 1.08, 0.77‐1.52; current smoker: OR = 1.77, 1.50‐2.07), alcohol intake status (former drinker: OR = 1.36, 1.01‐1.82), and high HsCRP levels (1‐3 mg/L:OR = 1.156, 1.00‐1.35; ≥3 mg/L:OR = 1.20, 1.02‐1.41) were positively associated with incident lung cancer risk, however, the inverse association was showed in the participants with higher BMI (overweight [24.5 ≤ BMI < 28.0 kg/m^2^]: OR = 0.83, 0.72‐0.95; obesity [BMI ≥28.0 kg/m^2^]: OR = 0.77, 0.62‐0.96), coal dust exposure (OR = 0.89, 0.78‐1.01), or with lower LDL‐C (≥120 mg/dL: OR = 0.76, 0.63‐0.92) (Table 2). In addition, age‐adjusted logistic regression showed a positive association of smoking duration and age started smoking with lung cancer risk. However, no association was found between the risk of lung cancer with smoking cessation time, family history of cancer, family history of lung cancer, abdominal obesity, FBG, BP, TC, TG, and HDL‐C (Table S1).

**TABLE 2:** Age and multivariable adjusted ORs and 95% CIs of the predictors with lung cancer risk, Kailuan study, 2006‐2015

Predictors	Case/control	Age‐adjusted OR 95% CI^a^	Coefficient	Multi‐adjusted OR 95% CI^b^
Age, years				
<45	55/37 447	1.00		1.00
45‐55	252/38 225	4.49 (3.35‐6.01)	1.461	4.36 (3.25‐5.86)
55‐65	334/29 048	7.82 (5.88‐10.41)	1.997	7.48 (5.60‐10.01)
≥65	343/16 793	13.90 (10.45‐18.48)	2.562	13.01 (9.77‐17.57)
*P*_trend_		<.001		<.001
Gender				
Female	91/25 604	1.00		1.00
Male	893/95 909	2.11 (1.70‐2.62)	0.567	1.77 (1.40‐2.23)
*P*		<.001		<.001
Smoking status				
Never	520/79 475	1.00		1.00
Former	45/3999	1.29 (0.95‐1.75)	0.134	1.08 (0.77‐1.52)
Current	419/38 039	1.93 (1.69‐2.20)	0.579	1.77 (1.50‐2.07)
*P*_trend_		<.001		<.001
Smoking pack‐years				
Never	520/79 475	1.00		1.00
<20	93/16 998	1.26 (1.07‐1.49)	0.284	1.34 (1,08‐1.66)
20‐40	168/14 402	1.72 (1.46‐2.02)	0.455	1.56 (1.32‐1.85)
≥40	1456/5894	2.60 (2.16‐3.12)	0.850	2.33 (1.92‐2.82)
*P*_trend_		<.001		<.001
Alcohol intake status				
Never	556/74 054	1.00		1.00
Former	58/3529	1.77 (1.35‐2.32)	0.281	1.36 (1.01‐1.82)
Current	370/43 930	1.39 (1.21‐1.58)	‐0.054	0.96 (0.82‐1.13)
*P*_trend_		<.001		.063
Coal dust exposure status				
Nonexposure	474/57 310	1.00		1.00
Exposure	510/64 203	1.07 (0.95‐1.22)	‐0.110	0.89 (0.78‐1.01)
*P*		.271		.082
BMI, kg/m^2^				
<18.5	35/3110	1.27 (0.90‐1.80)	0.177	1.20 (0.85‐1.70)
18.5‐23.9	497/57 981	1.00		1.00
24.0‐27.9	351/46 085	0.83 (0.72‐0.95)	‐0.189	0.83 (0.72‐0.95)
≥28.0	101/14 337	0.79 (0.64‐0.98)	‐0.223	0.77 (0.62‐0.96)
*P*_trend_		.008		.007
LDL‐C, mg/dL				
<70	273/26 031	1.00		1.00
70‐87	176/25 062	0.77 (0.63‐0.93)	‐0.264	0.76 (0.63‐0.92)
87‐100	173/23 853	0.83 (0.69‐1.01)	‐0.197	0.81 (0.67‐0.99)
100‐120	180/23 046	0.88 (0.72‐1.06)	‐0.158	0.84 (0.69‐1.02)
≥120	182/23 521	0.78 (0.64‐0.94)	‐0.298	0.72 (0.60‐0.88)
*P*_trend_		.032		.009
HsCRP, mg/L				
<1.0	427/62 813	1.00		1.00
1.0‐3.0	293/33 866	1.12 (0.96‐1.30)	0.141	1.16 (1.00‐1.35)
≥3.0	264/24 834	1.17 (1.00‐1.37)	0.184	1.20 (1.02‐1.41)
*P*_trend_		<.001		.045
Intercept			‐6.936	

Abbreviations: CI, confidence interval; OR, odd ratio.

^a^ Adjust for age class (<40, 40‐49, 50‐59, ≥60 y).

^b^ Multivariable logistic regression model was used with additional adjustment for all the other listed variables.

In the present study, we considered two set of models: the epidemiological model included six established predictors for lung cancer including age, gender, smoking status, alcohol intake status, coal dust exposure status, and BMI; and then through stepwise logistic regression, the full model additionally included two metabolic markers including HsCRP and LDL‐C (Table 2).

### 3.3 Predictive performance of the models

**FIGURE 1:**
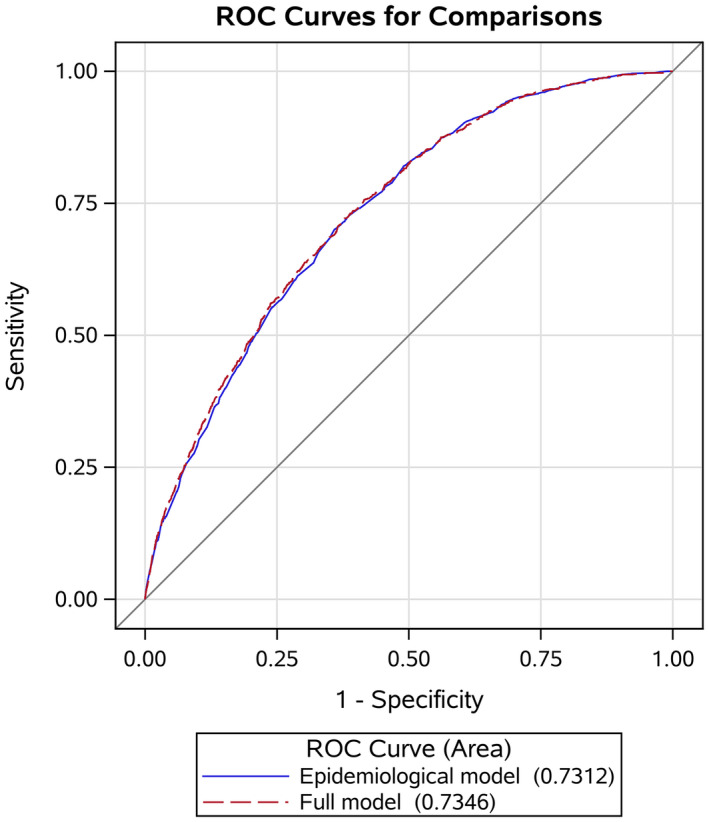
Receive operation curve for lung cancer risk prediction models, Kailuan study, 2006‐2015

The epidemiological risk prediction model generated a C‐statistic of 0.731. Significant improvement in C‐statistics was observed when the full model (C‐statistic = 0.735, *P* = .033) was compared to the epidemiological model (Table 3). ROC curves also suggested improved discrimination when adding metabolic markers to the epidemiological models (Figure 1). Stratified analysis by age showed that the discriminatory performance of the full model was better in participants <50 years (C‐statistic, 0.709) than in participants aged ≥50 years (C‐statistic, 0.655). Moreover, the full models yield better C‐statistic in females (C‐statistic, 0.726) than in males (C‐statistic, 0.716). Notably, the C‐statistic of the full model in former or current smokers (0.742) was higher than in never smokers and was statistically significantly higher than the C‐statistic of the epidemiological model in former or current smokers (0.735, *P* = .016) (Table 3).

**TABLE 3:** Predictive performance (C‐statistics) of the risk prediction models for lung cancer, Kailuan study, 2006‐2015

	Case/control	Epidemiological model^a^	Full model^b^	
		C‐statistics	C‐statistics	*P* value
Overall	984/121 513	0.729	0.735	.015
By age				
<50	142/52 783	0.709	0.709	.987
≥50	842/68 730	0.649	0.655	.012
By gender				
Female	91/25 604	0.730	0.726	.587
Male	893/95 909	0.717	0.716	.046
By smoking status				
Never	520/79 475	0.745	0.766	.093
Former or current	464/42 038	0.736	0.742	.107

Abbreviations: BMI, body mass index; HsCRP, high‐sensitivity C‐reactive protein; LDL‐C, low‐density lipoprotein cholesterol.

^a^ Epidemiological model: included age, gender, smoking status, smoking pack‐years, alcohol intake status, coal dust exposure status, and BMI.

^b^ Full model: additionally, included HsCRP and LDL‐C.

The results of internal validation by 10‐fold cross‐validation showed the stability of the models’ predictive power. The average C‐statistic of the epidemiological model and the full model were 0.728 and 0.735, respectively (Table S2).

Table S3 showed reclassification results. Compared with the epidemiological model, statistically significant (*P* < .001) higher NRI was observed for the full model (15.4, 95% CI, 9.1‐21.6). Similarly, we found statistically significant improvement for the IDI (*P* < .001) for the full model (0.03, 95% CI, 0.02‐0.05).

The full model showed good calibration across deciles of predicted risk (*P*_HL_ = .689). The predicted risk for lung cancer was 2.40% in the highest decile compared with 0.09% in the lowest decile (OR, 38.26; 95% CI, 18.95‐77.23) (Table 4). Meanwhile, the full model also showed good calibration in all subpopulations.

**TABLE 4:** Calibration of risk prediction models for lung cancer overall and by age across deciles of predicted risk, Kailuan study, 2006‐2015

	Decile of predicted risk of lung cancer										*P*_HL_ ^a^	OR (95% CI)^b^
	1	2	3	4	5	6	7	8	9	10		
Overall												
Observed	8	18	26	57	67	84	105	138	179	302		
Expected	10.4	16.1	25.8	44.3	65.7	89.5	110.8	138.0	187.6	295.7		
Observed risk	0.07%	0.15%	0.21%	0.47%	0.55%	0.69%	0.86%	1.13%	1.47%	2.45%		
Predicted risk	0.09%	0.13%	0.21%	0.36%	0.54%	0.73%	0.90%	1.13%	1.54%	2.40%	.689	38.26 (18.95‐77.23)
Age <50												
Observed	3	3	4	13	7	11	7	21	31	42		
Expected	3.3	4.2	5.6	7.7	8.1	9.2	12.6	17.0	28.4	45.9		
Observed risk	0.06	0.06	0.08	0.22	0.15	0.20	0.15	0.37	0.59	0.79		
Predicted risk	0.06	0.08	0.10	0.14	0.16	0.18	0.24	0.32	0.52	0.85	.353	14.22 (4.04‐45.89)
Age ≥50												
Observed	35	37	55	49	71	82	72	104	129	208		
Expected	27.0	37.8	48.2	57.4	65.5	76.8	91.6	109.7	131.7	196.5		
Observed risk	0.50	0.53	0.79	0.69	1.04	1.18	1.04	1.48	1.86	2.98		
Predicted risk	0.39	0.55	0.69	0.82	0.95	1.10	1.32	1.57	1.89	2.82	.217	6.08 (4.24‐8.71)
Female												
Observed	0	1	2	3	8	6	12	10	25	24		
Expected	0.5	1.0	1.6	3.7	7.8	9.3	10.3	12.3	17.8	26.7		
Observed risk	0.00%	0.04%	0.07%	0.04%	0.24%	0.35%	0.31%	0.43%	0.78%	1.30%		
Predicted risk	0.02%	0.04%	0.05%	0.07%	0.27%	0.34%	0.40%	0.46%	0.57%	1.35%	.659	NA
Male												
Observed	16	14	30	58	70	76	87	126	153	263		
Expected	11.8	17.1	27.1	48.9	67.4	80.9	100.1	126.4	160.5	252.8		
Observed risk	0.17%	0.15%	0.31%	0.60%	0.72%	0.81%	0.89%	1.33%	1.58%	2.66%		
Predicted risk	0.12%	0.18%	0.28%	0.51%	0.70%	0.86%	1.03%	1.31%	1.68%	2.56%	.536	16.55 (9.98‐27.44)
Never smoker												
Observed	4	7	15	33	33	49	70	77	98	134		
Expected	6.0	8.5	11.7	29.0	41.6	51.7	62.8	74.1	96.2	138.3		
Observed risk	0.05	0.09	0.19	0.41	0.41	0.60	0.89	0.98	1.26	1.62		
Predicted risk	0.07	0.11	0.14	0.36	0.52	0.65	0.79	0.95	1.21	1.70	.709	32.99 (12.19‐89.24)
Former or current smoker												
Observed	7	9	12	25	27	39	40	57	103	145		
Expected	7.1	9.0	11.7	23.1	30.3	36.2	47.0	63.1	86.5	150.1		
Observed risk	0.16	0.20	0.30	0.59	0.62	0.88	0.97	1.38	2.30	3.54		
Predicted risk	0.16	0.21	0.27	0.55	0.70	0.85	1.09	1.46	2.00	3.66	.670	23.00 (10.76‐49.13)

Abbreviations: CI, confidence interval; OR, odd ratio.

^a^ *P*_HL_: *P* value for Hosmer‐Lemeshow goodness‐of‐fit test; *P* > .05 indicates adequate fit.

^b^ OR of lung cancer for the top decile compared with the bottom decile of predicted prevalence.

To test the broad utility of our models for the LDCT screening set, in secondary analysis, we considered only participants aged more than 50 years old. As shown in Table S4, through stepwise regression, the included predictors and the corresponding associations were almost the same with the model developed among the whole population, which confirmed the stability and potential utility of our present models.

Finally, in the sensitivity analysis, if continuous variables were used instead of categorical variables, the C‐statistics of the full models were not improved (C‐statistic, 0.728).

## 4 DISCUSSION

In this study, we developed and validated internally two sets of risk prediction models for lung cancer based on data from routine health check‐ups, aiming at providing simple and efficient tools for tailored lung cancer screening by identity high‐risk subpopulations effectively. Our results showed that the model that included solely demographic information and lifestyle behavior information could strongly discriminate incident lung cancer cases from noncases. Moreover, the incorporation of CRP and LDL‐C as metabolic markers provided a satisfactory increase in discriminatory performance (C‐statistic for the full model, 0.735). Because all the indicators included in this model can be acquired easily from general clinical or screening sets, the potential of translating into use is great. Internal validation suggested the models may perform well regarding model discrimination when applied to other populations.

The evidence base for the included predictors is one of the important measurements of the validity of a risk prediction model. In this study, all the predictors have been shown associated with lung cancer risk. It has been proven that smoking is causally associated with the risk of lung cancer since the 1950s.^46^ Additionally, alcohol intake was shown to be related to elevated lung cancer risk.^47^, ^48^ Moreover, reduced risk for lung cancer has been indicated in men or women with higher levels of BMI.^49^, ^50^ Consistent with previous evidence from epidemiological studies, in this study, we observed the positive association of smoking, alcohol, and inverse association of BMI with lung cancer risk. As for the metabolic markers, we had reported the elevated lung cancer risk for participants with low LDL‐C.^46^ Furthermore, based on 20 population‐based cohort studies in the United States, Asia, Australia, and Europe, muller et al found that former and current smokers with higher hsCRP had an increased risk of lung cancer.^41^

In addition to credible predictors, a risk prediction model should also meet performance standards related to discrimination defined as the ability to distinguish lung cancer cases from controls, and calibration defined as the consistency between observed and predicted risk for lung cancer. There have been several lung cancer prediction models for the general population developed in different population.^51^ For study design, multiple case‐control studies (eg, Liverpool Lung Project [LLP] model), and cohorts or randomized trials (eg, Prostate, Lung, Colorectal, and Ovarian Cancer Screening Trial [PLCO]_m2014_ model)^52^ were used for the development of lung cancer risk prediction model. In terms of study population, never smokers (eg, EPIC model),^20^ or overall population (eg, LLPi model)^29^ were included for developing risk models. To our knowledge, this study is the only study assessing CRP and lipids directly to develop a lung cancer risk prediction model. It is hard to directly compare the discriminatory performance of risk prediction models as each was developed in different populations with varying baseline risks or lengths of follow‐up time. Nevertheless, each of the models’ discriminative ability was relatively similar, with C‐statistics ranges from 0.72 to 0.86. Our model showed comparable predictive performance compared with previous studies.

A major limitation of our study is that we were not able to validate the risk prediction model externally to assess the general applicability. However, the results of the internal validation suggest promisingly that this model will obtain well performance when applied to other populations. Another limitation is that because of the limited number of identified squamous cell carcinoma (SCC, n = 150), adenocarcinoma (AC, 143), and small cell lung carcinoma (SCLC, 71), we did not construct separate models for these two histologic types. However, the goal of our model is to apply in the screening setting, and previous studies indicate that many of the commonly cited risk factors for lung cancer are shared by different pathological types. Furthermore, the competing risks for death and/or development of other kinds of cancer were not corrected in present model, which may lead to potential bias in terms of the predictive accuracy of the models. Additionally, as the logistic regression model was used in this study, certain time interval predicted risk could not be calculated. Finally, information on lung function, asbestos exposure, history of pneumonia, and history of chronic obstructive pulmonary disease was not collected, so their roles in lung cancer risk prediction could not be evaluated in this study. Meanwhile, this study has its unique strengths. To the best of our knowledge, this is the first model that predicts lung cancer risk by assessing the CRP and lipids levels in a population‐based study. The present study provides a few advantages for the development of lung cancer prediction model, given the large sample size, which enables us to validate the prediction model in an independent subset of the population, as well as the detailed information from questionnaire and blood test, especially the comprehensive information which is easily available in general settings, are particularly important in the stratification of population for screening.

In conclusion, we developed and validated internally a risk prediction model for lung cancer that incorporates metabolic markers, based on data from Chinese residents. The model consisted of predictors that are readily available or easily accessible in general clinical or primary care settings showed satisfactory performance in terms of both discrimination and calibration. Therefore, this model could be used effectively as a practical tool to identify high‐risk individuals for tailored lung cancer screening.

## CONFLICT OF INTEREST

The authors declare no conflict of interest.

## AUTHORS’ CONTRIBUTIONS

Z Lyu, N Li, S Chen, G Wang, S Wu, M Dai, and J He were involved in conception and design. N Li, M Dai, and J He were involved in development of methodology. Z Lyu, X Feng, X Li, Y Wen, Z Yang, and Y Wang were involved in acquisition of data. Z Lyu, F Tan, J Li, H Chen, C Lin, J Ren, and J Shi were involved in analysis and interpretation of data. Z Lyu, N Li, M Dai, and J He were involved in writing, review, and/or revision of the manuscript. N Li, F Tan, J Li, H Chen, C Lin, J Ren, J Shi, M Dai, and J He were involved in administrative, technical, or material support. N Li, M Dai, and J He were involved in study supervision. All the authors approved the final version of the manuscript.

## ETHICS APPROVAL AND CONSENT TO PARTICIPATE

The study was conducted in accordance with the guidelines of the Helsinki Declaration and was approved by the Medical Ethics Committee of the Kailuan Medical Group, Kailuan Company. Written informed consent forms were obtained from all participants.

## Supporting information

Tables S1‐S4

## Data Availability

The datasets for this manuscript are not publicly available because all our data are under regulation of both the National Cancer Center of China and Kailuan Group. Requests to access the datasets should be directed to Jie He, hejie@cicams.ac.cn and Shouling Wu, drwusl@163.com.
