# Supplementary material for: Risk prediction model for lung cancer incorporating metabolic markers: Development and internal validation in a Chinese population
Source: Cancer Med. 2020 Apr 6;9(11):3983–94. doi: 10.1002/cam4.3025 (PMC7286442; doi:10.1002/cam4.3025)
Supplement: Supplementary file 1 — Tables S1‐S4 [file CAM4-9-3983-s001.docx]

| **Table S1.** Age-adjusted odds ratios (ORs) and 95% confidence intervals (CIs) of candidate predictors with lung cancer risk, Kailuan study, 2006-2015 | | |
| --- | --- | --- |
| **Variables ^†^** | **Case/Control** | **Age-adjusted OR 95%CI ^‡^** |
| **Smoking duration (years)** |  |  |
| Never smokers | 520/79475 | 1.00 |
| <15 | 30/6614 | 1.56 (1.07-2.28) |
| 15-30 | 132/17860 | 1.66 (1.36-2.02) |
| ≥30 | 302/17410 | 1.96 (1.70-2.27) |
| *P* _trend_ |  | <0.001 |
| **Age started smoking (years old )** |  |  |
| Never smokers | 520/79475 | 1.00 |
| <20 | 171/15695 | 1.91 (1.60-2.27) |
| ≥20 | 293/26343 | 1.79 (1.55-2.07) |
| *P* _trend_ |  | 0.525 |
| **Smoking cessation duration (years)** |  |  |
| Never smokers | 520/79475 | 1.00 |
| <15 | 35/3179 | 1.36 (0.97-1.93) |
| ≥15 | 10/820 | 1.20 (0.64-2.26) |
| *P* _trend_ |  | 0.855 |
| **Family history of cancer** |  |  |
| No | 802/86786 | 1.00 |
| Yes | 36/3415 | 1.10 (0.79-1.54) |
| *P* |  | 0.580 |
| **Family history of lung cancer** |  |  |
| No | 824/89035 | 1.00 |
| Yes | 14/1166 | 1.27 (0.75-2.17) |
| *P* |  | 0.378 |
| **Abdominal obesity** |  |  |
| No | 539/65811 | 1.00 |
| Yes | 441/55137 | 0.81 (0.71-0.92) |
| *P* |  | 0.001 |
| **FBG (mmol/L)** |  |  |
| <3.9 | 40/3228 | 1.29 (0.94-1.78) |
| 3.9 – 5.6 | 746/98146 | 1.00 |
| 5.6 – 7.0 | 99/10191 | 1.10 (0.89-1.36) |
| ≥7.0 | 99/9935 | 0.98 (0.80-1.21) |
| *P* _trend_ |  | 0.755 |
| **Blood pressure (mm Hg)** |  |  |
| Low | 18/3773 | 0.76 (0.47-1.21) |
| Normal | 499/68355 | 1.00 |
| High | 464/49219 | 0.90 (0.79-1.20) |
| *P* _trend_ |  | 0.160 |
| **TC (mg/dL)** |  |  |
| <160 | 195/24425 | 1.00 |
| 160 - 180 | 174/23836 | 0.86 (0.70-1.05) |
| 180 - 198 | 197/24457 | 0.92 (0.75-1.12) |
| 198 - 220 | 197/25102 | 0.84 (0.69-1.03) |
| ≥220 | 221/23687 | 0.96 (0.79-1.16) |
| *P* _trend_ |  | 0.465 |
| **TG (mg/dL)** |  |  |
| <75 | 209/25695 | 1.00 |
| 75 - 100 | 196/24816 | 0.87 (0.72-1.06) |
| 100 - 133 | 198/24611 | 0.88 (0.72-1.07) |
| 133 - 200 | 198/23993 | 0.89 (0.73-1.09) |
| ≥200 | 183/22383 | 0.97 (0.80-1.19) |
| *P* _trend_ |  | 0.773 |
| **HDL-C (mg/dL)** |  |  |
| <46 | 178/21665 | 1.00 |
| 46 - 53 | 197/23785 | 1.01 (0.82-1.24) |
| 53 - 60 | 174/23646 | 0.89 (0.72-1.10) |
| 60 - 69 | 186/25640 | 0.85 (0.69-1.05) |
| ≥69 | 248/26778 | 0.94 (0.77-1.14) |
| *P* _trend_ |  | 0.256 |
| Abbreviations: OR: odds ratio; CI: confidence interval; FBG: fasting blood glucose; TC: total cholesterol; TG: triglycerides; HDL-C: high-density lipoprotein cholesterol.  **^†^** These variables have missing values. **^‡^** Adjust for age class (<40, 40-49, 50-59, ≥60 years). | | |

| **Table S2.** Predictive performance **(**C-statistics) of the risk prediction models in internal validation, Kailuan study, 2006-2015 | |
| --- | --- |
| Sub-dataset | C-statistics |
| **Epidemiological Model ^†^** |  |
| Sub-dataset 1 | 0.757 |
| Sub-dataset 2 | 0.748 |
| Sub-dataset 3 | 0.728 |
| Sub-dataset 4 | 0.730 |
| Sub-dataset 5 | 0.733 |
| Sub-dataset 6 | 0.712 |
| Sub-dataset 7 | 0.724 |
| Sub-dataset 8 | 0.725 |
| Sub-dataset 9 | 0.711 |
| Sub-dataset 10 | 0.714 |
| **Average** | **0.728** |
| **Full Model ^‡^** |  |
| Sub-dataset 1 | 0.716 |
| Sub-dataset 2 | 0.760 |
| Sub-dataset 3 | 0.749 |
| Sub-dataset 4 | 0.723 |
| Sub-dataset 5 | 0.737 |
| Sub-dataset 6 | 0.734 |
| Sub-dataset 7 | 0.773 |
| Sub-dataset 8 | 0.722 |
| Sub-dataset 9 | 0.723 |
| Sub-dataset 10 | 0.716 |
| **Average** | **0.735** |
| ^†^ Epidemiological model: including age, gender, smoking status, smoking pack-years, alcohol intake status, coal dust exposure status, and BMI.  ^‡^ Full model: further included hsCRP and LDL-C. | |

| **Table S3.** Net reclassification improvement (NRI) and integrated discrimination improvement (IDI) of the risk prediction models for lung cancer, Kailuan study, 2006-2015 | | | |
| --- | --- | --- | --- |
|  | Epidemiological Model ^†^ | Full Model ^‡^ | |
|  | Values | Values | *P value* |
| **NRI (95% CI)** |  |  |  |
| Category-free NRI (%) | Ref | 15.4 (9.1-21.6) | <0.001 |
| % of events correctly reclassified | - | 0 |  |
| % of non-events correctly reclassified | - | 16 |  |
| **IDI (95% CI)** | Ref | 0.03 (0.02-0.05) | <0.001 |
| ^†^ Epidemiological model: including age, gender, smoking status, smoking pack-years, alcohol intake status, coal dust exposure status, and BMI.  ^‡^ Full model: further included hsCRP and LDL-C. | | | |

| **Table S4.** Age and multi-variable adjusted odds ratios (ORs) and 95% confidence intervals (CIs) of the predictors with lung cancer risk among participants age ≥ years, Kailuan study, 2006-2015 | | | | |
| --- | --- | --- | --- | --- |
| **Predictors** | **Case/Control** | **Age-adjusted**  **OR 95%CI ^†^** | **Coefficient** | **Multi-adjusted**  **OR 95%C ^‡^** |
| **Age, years** |  |  |  |  |
| 50-55 | 165/22889 | 1.00 |  | 1.00 |
| 55 - 60 | 200/19213 | 1.44 (1.17-1.78) | 0.380 | 1.46 (1.19-1.80) |
| 60 - 65 | 134/9835 | 1.89 (1.50-2.38) | 0.642 | 1.90 (1.51-2.40) |
| 65 - 70 | 171/7461 | 3.18 (2.56-3.940 | 1.174 | 3.24 (2.60-4.03) |
| ≥70 | 172/9322 | 2.56 92.06-3.17) | 0.941 | 2.56 (2.05-3.20) |
| *P* _trend_ |  | <0.001 |  | <0.001 |
| **Gender** |  |  |  |  |
| Female | 70/11631 | 1.00 |  | 1.00 |
| Male | 772/57099 | 2.09 (1.63-2.67) | 0.520 | 1.68 (1.30-2.18) |
| *P* |  | <0.001 |  | <0.001 |
| **Smoking status** |  |  |  |  |
| Never | 444/45242 | 1.00 |  | 1.00 |
| Former | 44/2917 | 1.36 (1.00-1.86) | 0.162 | 1.18 (0.84-1.65) |
| Current | 354/20571 | 1.99 (1.72-2.29) | 0.567 | 1.76 (1.48-2.09) |
| *P* _trend_ |  | <0.001 |  | <0.001 |
| **Smoking pack-years** |  |  |  |  |
| Never | 444/45242 | 1.00 |  | 1.00 |
| <20 | 88/6911 | 1.57 (1.24-1.98) | 0.397 | 1.49 (1.17-1.89) |
| 20-40 | 172/11402 | 1.70 (1.42-2.03) | 0.455 | 1.58 (1.31-1.89) |
| ≥40 | 138/5151 | 2.55 (2.10-3.09) | 0.832 | 2.30 (1.88-2.81) |
| *P* _trend_ |  | <.001 |  | <0.001 |
| **Alcohol intake status** |  |  |  |  |
| Never | 471/44242 | 1.00 |  | 1.00 |
| Former | 53/2587 | 1.80 (1.35-2.40) | 0.314 | 1.37 (1.10-1.87) |
| Current | 318/21901 | 1.50 (1.30-1.74) | 0.054 | 1.06 (0.89-1.26) |
| *P* _trend_ |  | <0.001 |  | 0.141 |
| **Coal dust exposure status** |  |  |  |  |
| Non-exposure | 416/34188 | 1.00 |  | 1.00 |
| Exposure | 426/34542 | 1.06 (0.93-1.22) | -0.122 | 0.89 (0.77-1.02) |
| *P* |  | 0.271 |  | 0.090 |
| **BMI, kg/m^2^** |  |  |  |  |
| <18.5 | 28/1516 | 1.14 (0.77-1.67) | 0.082 | 1.09 (0.74-1.60) |
| 18.5 – 23.9 | 434/31245 | 1.00 |  | 1.00 |
| 24.0 – 27.9 | 298/27682 | 0.79 (0.68-0.91) | -0.234 | 0.79 (0.68-0.92) |
| ≥28.0 | 82/8287 | 0.72 (0.57-0.91) | -0.31 | 0.74 (0.58-0.94) |
| *P* _trend_ |  | <0.001 |  | 0.004 |
| **LDL-C, mg/dL** |  |  |  |  |
| <70 | 247/16053 | 1.00 |  | 1.00 |
| 70 - 87 | 143/13515 | 0.72 (0.59-0.89) | -0.320 | 0.73 (0.59-0.90) |
| 87 - 100 | 142/12369 | 0.81 (0.65-0.99) | -0.220 | 0.80 (0.65-0.99) |
| 100 - 120 | 152/12338 | 0.87 (0.71-1.06) | -0.151 | 0.86 (0.70-1.06) |
| ≥120 | 158/14455 | 0.76 (0.62-0.93) | -0.315 | 0.73 (0.60-0.89) |
| *P* _trend_ |  | 0.156 |  | 0.009 |
| **HsCRP, mg/L** |  |  |  |  |
| <1.0 | 349/31936 | 1.00 |  | 1.00 |
| 1.0 – 3.0 | 257/19759 | 1.13 (0.96-1.33) | 0.168 | 1.18 (1.00-1.39) |
| ≥3.0 | 236/17035 | 1.15 (0.97-1.36) | 0.173 | 1.19 (1.00-1.41) |
| *P* _trend_ |  | <0.001 |  | 0.059 |
| **Intercept** |  |  | -5.369 |  |
| Abbreviations: OR: odd ratio; CI: confidence interval; BMI: body mass index; LDL-C: low-density lipoprotein cholesterol; HsCRP: high sensitivity C-reactive protein.  ^†^ Adjust for age class (50-55, 55-60, 60-65, 65-70, ≥70 years).  **^‡^** Additionally adjusting for all the other listed variables. | | | | |
